# 2224: Characterizing vigilant thoughts and behaviors that disrupt sleep in veterans and utilization of cognitive techniques

**DOI:** 10.1017/cts.2017.122

**Published:** 2018-05-10

**Authors:** Mary Katherine Howell, Thomas Mellman

**Affiliations:** Georgetown - Howard Universities, Washington, DC, USA

## Abstract

OBJECTIVES/SPECIFIC AIMS: Sleep disturbance is a common problem following military deployment. Insomnia is associated with other adverse psychiatric and medical health outcomes. There are specialized cognitive behavioral therapies that can effectively treat insomnia; however, these tend to emphasize dysfunctional beliefs about sleep rather than nocturnal vigilance. Deployment to a threatening environment can engender nocturnal vigilance, which appears to be a salient feature of sleep disturbance in formerly deployed veterans. The purpose of this analysis is to characterize sleep-interfering thoughts and behaviors observed in an ongoing pilot study of a novel 2-session intervention incorporating various cognitive techniques to improve sleep in veterans. METHODS/STUDY POPULATION: To date, 10 formerly deployed US veterans with disturbed sleep have been recruited from the greater DC area. Participants are assessed at baseline, receive 2 intervention sessions, and are again assessed in 3 months. Sleep-interfering thoughts and behaviors are evaluated via self-report forms including the Fear of Sleep Inventory (FoSI), interviews, and prospective diaries. A portion of both intervention sessions addresses vigilant behaviors and sleep-interfering thoughts by teaching participants 1 of 4 techniques that target nocturnal vigilance: cognitive defusion, body scan, self-guided pleasant imagery, and dream rescripting. RESULTS/ANTICIPATED RESULTS: All of the first 10 participants endorsed sleep-interfering thoughts on the Fear of Sleep Index (FOSI) at a severity level of at least “a few times per month” (rating of ≥1), including several regarding previous trauma (#5) and nightmares (#10 and #16). Other elicited thoughts included thoughts about their environment (n=6), sleep (n=5), social or occupational concerns (n=8), nightmares (n=5), and health (n=4). All of the first 10 participants endorsed vigilant behaviors, including being over-attentive to their environment (n=7), checking behaviors (n=6), and being “on-guard” (n=8). Cognitive technique was selected by the participant in collaboration with the facilitator. Customized recommendations were given as to the timing and duration of practice, but all participants were instructed to practice at least once daily. Three participants (n=3) were fully compliant with their cognitive technique recommendations (choosing a body scan or imagery), 5 were partially compliant, and 2 were not compliant (both chose cognitive defusion). There was a significant reduction in sleep onset latency and wake after sleep onset from baseline to post-treatment (*p*<0.05). DISCUSSION/SIGNIFICANCE OF IMPACT: The preliminary data suggests that veterans exhibit cognitive and behavioral patterns that involve vigilance and interfere with sleep and demonstrates the need for an intervention targeting the link between nocturnal vigilance and sleep disturbance. More veteran participants and feedback are needed to optimize the efficacy and effectiveness of this sleep training.

